# Bioinspired Multi‐Stimuli Responsive Actuators with Synergistic Color‐ and Morphing‐Change Abilities

**DOI:** 10.1002/advs.202101295

**Published:** 2021-06-10

**Authors:** Xinkai Li, Jize Liu, Dongdong Li, Shaoquan Huang, Kai Huang, Xinxing Zhang

**Affiliations:** ^1^ State Key Laboratory of Polymer Materials Engineering Polymer Research Institute of Sichuan University No.24 South Section 1, Yihuan Road Chengdu 610065 China; ^2^ National Engineering Research Center for Non‐Food Biorefinery Guangxi Key Laboratory of Bio‐refinery Guangxi Academy of Sciences 98 Daling Road Nanning 530007 China; ^3^ Environmental protection facilities or service department Guangxi Beitou Environmental Protection & Water Group Co. Ltd. 153 Minzu Avenue Nanning 530029 China

**Keywords:** actuators, cellulose nanocrystals, color‐changing, infrared/humidity sensitive, multi‐stimuli responses

## Abstract

The combination of complex perception, defense, and camouflage mechanisms is a pivotal instinctive ability that equips organisms with survival advantages. The simulations of such fascinating multi‐stimuli responsiveness, including thigmotropism, bioluminescence, color‐changing ability, and so on, are of great significance for scientists to develop novel biomimetic smart materials. However, most biomimetic color‐changing or luminescence materials can only realize a single stimulus‐response, hence the design and fabrication of multi‐stimuli responsive materials with synergistic color‐changing are still on the way. Here, a bioinspired multi‐stimuli responsive actuator with color‐ and morphing‐change abilities is developed by taking advantage of the assembled cellulose nanocrystals‐based cholesteric liquid crystal structure and its water/temperature response behaviors. The actuator exhibits superfast, reversible bi‐directional humidity and near‐infrared (NIR) light actuating ability (humidity: 9 s; NIR light: 16 s), accompanying with synergistic iridescent appearance which provides a visual cue for the movement of actuators. This work paves the way for biomimetic multi‐stimuli responsive materials and will have a wide range of applications such as optical anti‐counterfeiting devices, information storage materials, and smart soft robots.

## Graphical abstract

A delicately designed intelligent actuator is presented to exhibit superfast, reversible bi‐directional humidity/near‐infrared light actuating abilities and synergistic iridescent appearance. Taking advantage of the self‐assembled cellulose nanocrystals‐based cholesteric liquid crystal structure and its water/temperature response behaviors, the simulations of the butterflies’ vivid structural color are successfully realized.

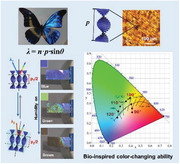

## 1 Introduction

Natural organisms own the ability to respond to external stimuli, which are ingenious designs of nature.^[^ ^1^ ^]^ Specifically, some kinds of animals can respond immediately in the form of color‐changing,^[^ ^2^ ^]^ deformation,^[^ ^3^, ^4^, ^5^, ^6^ ^]^ or bioluminescence^[^ ^7^ ^]^ to avoid damage from an outside force. For instance, chameleons and cephalopods can take on the colors of the environment nearby to avoid detection. In addition, butterfly wings and beetle's exoskeleton often display vivid and bright structural colors which change with their movement and direction changing of the incident light.^[^ ^8^, ^9^ ^]^ Such characteristic brightness is derived from interference, diffraction grating, or light scattering of periodically layered or lattice nanostructures, which provides a high refractive index and broadband light absorption.^[^ ^10^ ^]^ Therefore, intelligent materials integrating with structural colors of butterfly wings and multi‐stimuli responsiveness, will have broad application prospects in the next generation of flexible electronics.

Recently, several bio‐inspired materials with color‐changing ability have been developed in a variety of shapes and structures with sophisticated functions.^[^ ^11^, ^12^, ^13^, ^14^, ^15^, ^16^, ^17^, ^18^ ^]^ Ko et al. developed an electrothermal soft actuator with active color‐changing functions through thermochromic pigment.^[^ ^19^ ^]^ Yang's group prepared a humidity‐responsive film showing the synergistic shape and fluorescence color change based on bimorph strategy.^[^ ^20^ ^]^ Zhao et al. presented a bilayer structural photonic crystal hydrogel actuator based on a hybrid inverse opal scaffold, which can demonstrate a reversible coloration switch during the structural change.^[^ ^21^ ^]^ Although these studies have achieved satisfying results, in most cases, only a single stimulus‐responsive actuation function can be achieved. So far, there are few intelligent materials that can realize multiple stimulus responses and simulation of butterflies’ brilliant structural colors at the same time.

As a renewable material extracted from natural sources, cellulose nanocrystals (CNCs) are rod‐like crystalline residues with abundant free hydroxyl groups, which are usually produced by acid hydrolysis of natural cellulose.^[^ ^22^, ^23^ ^]^ Room‐temperature dried CNCs suspension can form an iridescent cholesteric liquid crystal (LC) film with a left‐handed helical structure. Similar to beetle's exoskeleton, such solid CNCs films can selectively reflect incident light and display variable structural color, which depends on the incident angle and the cholesteric pitch.^[^ ^24^, ^25^, ^26^ ^]^ Nevertheless, the assembly process and the resulted LC structures of CNCs are still hard to control. How to uniformly distribute CNCs on the surface of elastomer and integrate them into flexible intelligent materials are still formidable tasks.

Here, a humidity/infrared‐sensitive intelligent actuator with synergistic structural color changes is developed based on the self‐assembled CNCs film and polyurethane (PU) substrate. To prevent the outward radial capillary flow and Marangoni flow during the evaporation process of CNCs, glucose is added as a thickener to regulate the assembly behavior of CNCs and facilitate the formation of a uniform cholesteric LC structure. In addition, in situ synthesized silver nanoparticles (AgNPs) are dispersed into the PU matrix to endow the elastomer with excellent photothermal conversion efficiency and thermal conductivity. The cholesteric LC display, excellent hydroscopicity, and extremely low coefficient of thermal expansion (CTE) (< 5×10^−6^ K^−1^, at least one order of magnitude lower than most metals, ceramics, and plastics) of the assembled CNCs film endow the actuator with alterable schemochrome and reversible humidity/infrared actuating ability, successfully realizing the simulation of animal skins. The strategy provided here will open up a new way to develop various multi‐stimuli responsive materials with color‐ and morphing‐change abilities, which is promising to broad application prospects in the fields of anti‐counterfeiting, information storage, and soft robots.

## 2 Results and Discussion

**Figure 1:**
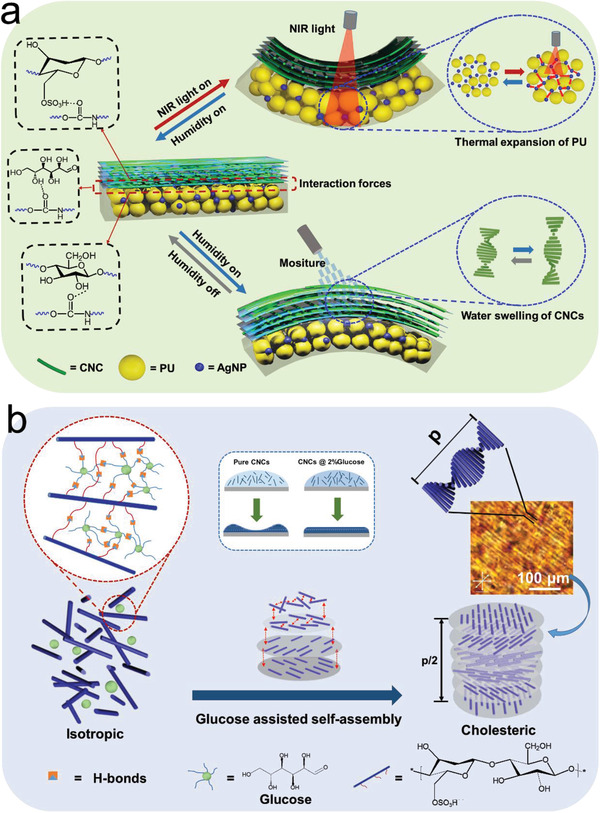
a) Design of the bio‐inspired bilayer composite film based on CNCs and its NIR light‐ and humidity‐responsive actuating principles. b) Schematic of the glucose‐assisted self‐assembly process of CNCs/glucose suspension and the POM image of a typical fingerprint pattern of the cholesteric phase.

The integration of multi‐stimuli responsiveness of actuation deformation with vivid structural color‐changing ability into one single material system is of great significance to the development of smart biomimetic materials. Inspired by environmentally sensitive animal skin and the butterfly wings’ alterable schemochrome, we develop a bilayer film consists of self‐assembled CNCs cholesteric LC layer and photothermal conversion PU elastomer layer to simultaneously realize fascinating multi‐functional properties, including reversible bi‐directional humidity‐ and near‐infrared (NIR) light‐actuating ability and synergistic iridescent appearance (**Figure** **1**a,b).

In the bilayer structure described above, the soft PU elastomer layer was designed as the platform for self‐assembly of CNCs to provide the material with flexibility. Due to the hydrogen bonds formed during the self‐assembly process and interactions of polar groups, the PU layer and the CNCs layer can cling to each other tightly (Figure 1a).^[^ ^27^ ^]^ AgNPs possessing high photothermal conversion efficiency are added to ensure the desirable photothermal effects of the elastomer layer.^[^ ^28^ ^]^ The mismatch in CTE and water absorption between PU and CNCs provide the material with satisfying NIR‐ and humidity‐actuating performance.

The upper layer is the iridescent cholesteric LC layer based on CNCs, which provides the material with unique optical properties, is designed as shown in Figure 1b. During the gradual evaporation of water at room temperature, the outward radial capillary flow and Marangoni flow are serious obstacles to the formation of self‐assembled CNCs‐based cholesteric LC structure.^[^ ^29^, ^30^ ^]^ The problems described above can be solved by adding a proper percentage of glucose (2%), which intercalate into the original spiral‐shaped microstructure via interstitial volumes within nanocrystals and between nematic monolayers. Working as a thickener, glucose has effectively increased the viscosity and reduced both capillary flow and Marangoni flow without interfering with CNCs twisted assembly. The strategy proposed is favorable for solving the problem that it is difficult to build a uniform LC structure layer on the surface of the polymer matrix.

**Figure 2:**
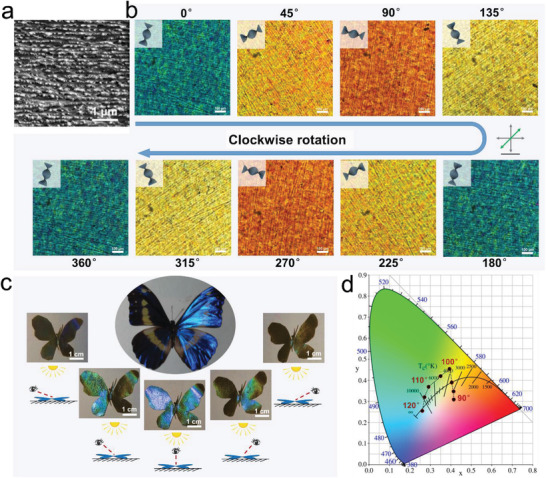
Optical properties of the CNCs@glucose layer. a) SEM cross‐section image of the film showing evident cholesteric structure of CNCs. b) POM‐*λ* images of the film showing a periodic color change from blue to orange following the clockwise rotation. c) The angle‐related structural colors of an artificial “Morpho Helena”. d) CIE 1931 color space chromaticity diagrams for the LC layer during the color‐changing process, coordinate values of which were obtained for every 5° increment in the view angle.

The microstructure of a dried CNCs@glucose film is characterized by scanning electron microscopy (SEM) (**Figure** **2**a and Figure S1, Supporting Information). In the gradual evaporation of water, the CNCs realize a first‐order phase transition from isotropic to a cholesteric phase and finally form an LC film.^[^ ^31^, ^32^, ^33^, ^34^ ^]^ As the polarized optical microscopy (POM) image shows, the film owns a typical fingerprint pattern of the cholesteric phase. The multilayered structure and left‐handed twisting rod morphology of a chiral nematic phase can be obviously observed in the cross‐section image, confirming the cholesteric LC architecture of the film. The cholesteric LC molecules are parallel to each other and arranged into layers. The long axis direction of the molecules in different layers changes slightly, and they are arranged into a helical structure along the normal direction. Such unique ordered phase of cholesteric LC owns a high birefringence and its characteristic fingerprint pattern can be easily observed in POM. The spiral arrangement of the LC molecular layer will cause periodic changes in the optical refractive index, resulting in periodic birefringent extinction fringes in the fingerprint texture.^[^ ^35^, ^36^, ^37^ ^]^ The spacing of the fringes is equal to half the pitch (*p*/2). The wavelength *λ* of the selectively reflected light and the pitch *p* of the cholesteric LC material can be described by Bragg's reflection equation of crystal diffraction:

(1) λ=n·p·sinϑ

Where *n* is the average refractive index of the sample and *θ* is the angle of incidence.^[^ ^38^ ^]^ As shown in Figure 2b, the special optical appearance of the film changes with rotation angle. The POM‐*λ* images of the LC layer show alternating light and dark stripes, and exhibit a periodic color change from blue to yellow to orange following the clockwise rotation. Due to the cholesteric LC molecules formed a left‐handed helix structure, when the cholesteric pitch of CNCs is close to a certain wavelength, the Bragg scattering of light of the corresponding wavelength will be caused, and the corresponding color will be presented.

The selective reflection properties of the CNCs‐based cholesteric LCs can be easily observed by naked eyes. As the viewing angle increases from 90° to 120°, the structure color covers a wide color range from brown to green to blue (Figure S2, Supporting Information). Based on this color‐changing property, an artificial “Morpho Helena” is shaped to imitate the vivid structural color of this kind of pretty butterfly (Figure 2c). In addition, to further understand the property of the LC layer, a colorimeter was used to quantitatively characterize the above property. The colors of the film at different view angles were measured from 90° to 120° with a 5° interval (Figure 2d). The *x* and *y* coordinates shifted from brown to green to blue with increasing view angle. The radar image in Figure S3, Supporting Information shows that the reading “*a*” (indicate the range from magenta to green) and “*b*” (indicate the range from yellow to blue) is negatively correlated with the included angle between the instrument and the film, which lays a foundation for this material to realize actuation and discoloration simultaneously.

**Figure 3:**
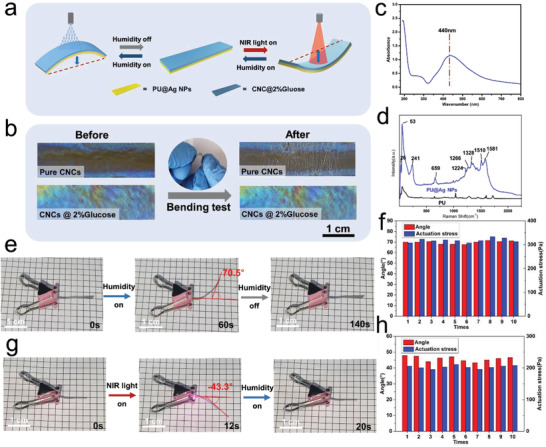
NIR‐ and humidity‐actuating performance of the material. a) Schematic illustration of a bilayer smart gripper bending on exposure to NIR/humidity. b) Optical images of PU/CNC bilayer film and PU/CNC@glucose bilayer film before and after bending for 5000 times. c) UV‐visible spectrum and d) Laser confocal Raman microspectroscopy of the material. Photographs of an actuator reversibly bending when e) humidity and g) NIR light is alternately switched on and off. Bending angle and actuation force of the film with f) humidity and h) NIR light alternately turned on and off.

As shown in **Figure** **3**a, the bilayer composite film shows reversible bi‐directional actuation activated by NIR light and relative humidity changes. Long‐term structural reliability and a certain amount of flexibility are indispensable prerequisites for the practical application of soft actuators. Nevertheless, during the self‐assembly process, the surface tension gradient induced by the variation of evaporation flux across the surface causes the outward radial capillary flow and Marangoni flow. These two factors will lead to the accumulation of large‐sized CNCs nanorods at the perimeter and the formation of a ring‐shaped deposition, which will harmfully induce shear alignment and further disrupt the cholesteric sequence.

The coffee‐ring effect described above can be solved by adding glucose, which intercalates into the original spiral‐shaped microstructure via interstitial volumes within nanocrystals and between nematic monolayers. Working as a thickener, glucose has effectively increased the viscosity and reduced both capillary flow and Marangoni flow without interfering with CNCs twisted assembly. As shown in Figure 3b, the CNCs@glucose layer can uniformly distribute and reach a flat shape across the substrate, whereas pure CNCs would not. More importantly, the glucose‐assisted self‐assembled CNCs film based actuator maintains a great optical appearance without cracks even after 5000 bending cycles, which should give the credit to the hydrogen bond network formed between glucose and CNCs.^[^ ^39^, ^40^ ^]^ In contrast, in the case of the control sample based on pristine CNCs, hundreds of bending cycles will cause serried cracks in the CNCs layer, which will seriously damage the optical appearance of the material. Therefore, the strategy we proposed is able to equip bilayer actuators with long‐term reliability and enough flexibility to support their stable actuation performance.

Thanks to its abundant free hydroxyl groups, the self‐assembled CNC layer owns excellent water absorption and expansion capacity.^[^ ^41^, ^42^, ^43^ ^]^ In consequence, when the actuator film is exposed to humidity, it will bend toward the non‐absorbent PU layer with large actuation amplitude. The experimental result (Figure 3e) shows that the horizontal bending angle of the actuator increases from 0° to 70.5° as the moisture absorption time increases from 0 to 60 s, and then it takes 80 s to revert to its original position without humidifying. Besides, the humidity‐actuation in the vertical orientation is also feasible. The vertical bending angle of the actuator increases from 0° to 144.2° as the moisture absorption time increases from 0 to 14 s, and then it takes 71 s to revert to its original position without humidifying (Figure S4, Supporting Information). In addition, we have studied the effect of original ambient relative humidity (RH_0_) on the humidity‐actuating property of the material at room temperature. The results (Figure S5, Supporting Information) indicate that the effect of RH_0_ on the humidity‐actuating property is not significant, so that the actuator can work steadily in different kinds of weather conditions.

In this study, AgNPs were prepared by in situ reduction of silver ammonia by glucose. As a reductant with abundant hydroxyl groups, glucose enables AgNPs to homogeneously disperse in aqueous systems including water and PU latex (Figure S6, Supporting Information). Laser confocal Raman microspectroscopy, Ultraviolet‐visible (UV‐visible) spectrometer, and SEM were used to demonstrate the successful synthesis of AgNPs. As shown in Figure S7, Supporting Information), the diameter of the in situ synthesized AgNPs is about 221±25 nm. Moreover, the peak around 440 nm in the UV‐visible spectrum of the sample (Figure 3c) is attributed to the AgNPs. Raman spectra had been obtained for pure PU and PU/AgNPs composites. As can be analyzed and compared from results presented in Figure 3d, the signal at 26, 53, 241, 659, 1224, 1266, 1328, 1510, and 1581 cm^−1^ can be attributed to the AgNPs dispersed in the PU elastomer. It has been proved that AgNPs acting as a photothermal agent can effectively and controllably convert 808 nm NIR light into heat. The addition of AgNPs endows the sample with high photothermal conversion efficiency and thermal conductive properties. Taking advantage of a great mismatch in the coefficient of CTE between the PU layer and the CNCs layer, the sample shows satisfactory NIR actuating performance. As shown in Figure 3g and Movie S1, Supporting Information, when the actuator film is exposed to NIR light, it takes 12 s to bend to maximum angle (−43.3°) for the strip‐shaped sample. The NIR‐actuation direction of the sample is completely opposite to humidity‐actuation, which endow the actuator with bi‐directional actuate capability. The repetitive experimental results (Figure 3f,h; Figures S8 and S9, Supporting Information) have proved that the actuator owns great stability and repeatability, which can be turned on and off many times and maintain steady actuating speed and force. Besides, we have evaluated the durability of the constructed actuator. As shown in Figure S10, Supporting Information, after subjected to 5000 bending cycles, the actuator still exhibited stable actuating performance, revealing excellent structural stability.

**Figure 4:**
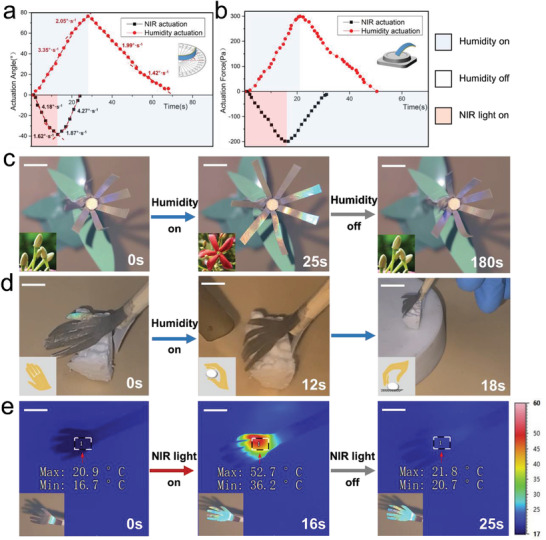
The comparison of the two actuating methods (humidity and NIR) about actuation features including a) speed and b) force. Digital images of c) a smart “rangoon creeper flower” blooming and closing. d) A “palm” grasps and transfers an object to the destination. e) A “palm” changes its color while bending its fingers and the temperature variation during the process. Scale bars, 1 cm.

**Figure 5:**
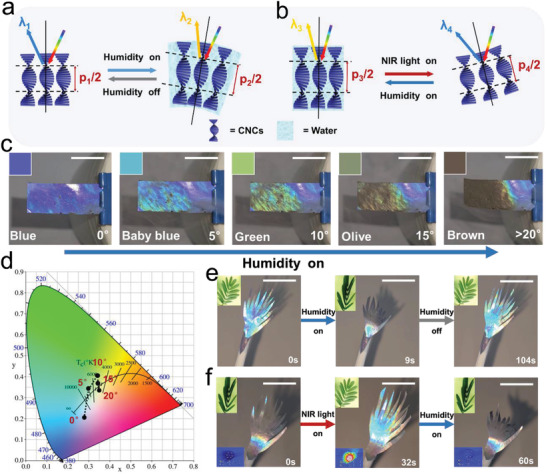
The mechanism of structural color changing of CNCs during the a) humidity and b) NIR laser light actuating process. c) Digital pictures and d) CIE 1931 color space chromaticity diagrams show the relationship between color changing and actuating angle. Photographs of a smart “mimosa” splaying and closing when exposed to e) moisture and f) NIR light. Scale bars, 1 cm.

The actuation features of the two actuating methods have been studied and compared. As shown in **Figure** **4**a,b, the NIR‐actuating process exhibits an actuating speed up to 4.18° s^−1^, which is higher than that of the humidity‐actuating process (3.35° s^−1^). However, the humidity‐actuating process exhibits greater actuation force (299.4 Pa) and can reach to larger actuation angle (76.5°) than these of the NIR‐actuating process (199.1 Pa and 38.4°). In addition, the actuating direction of the two actuating methods is in completely opposite ways so that the actuator can achieve two ways of color‐changing (blue‐brown‐blue or brown‐blue‐brown) as shown in **Figure** **5**e,f. In consequence, two different options are provided, and we can choose the more suitable one based on required actuation features including direction, speed, strain, and color‐changing pattern. We developed a series of bionic motion robots based on the abovementioned bi‐directional actuation capability of the material under humidity/NIR laser stimulation. Herein the NIR intensity employed is 1.2 W cm^−2^. Inspired by the color‐changing behavior (from white to red) during florescence of the rangoon creeper, a biomimetic “flower” is shaped which can undergo repeated closing and blooming in response to humidity on and off respectively (Figure 4c and Movie S2, Supporting Information). In the meantime, the “florescence” process is accompanied by periodic structure color changing from claybank to blue. In addition, utilizing the satisfactory actuating force, the film can be shaped to a specified shape (such as the geometry of a hand) and perform some physical work. As shown in Figure 4d, Movies S3 and S4, Supporting Information, when exposed to moisture, the artificial “palm” can smoothly bend its fingers to grasp and transfer an object. In contrast, an NIR laser can uncurl the fingers of the “palm” in a short moment (12 s). The temperature and the color variation during the process are shown in Figure 4e, Figures S11 and Movie S5, Supporting Information. The working condition of the actuation can be easily recognized by the vivid structure color. The easily prepared device with reversible actuation ability and alterable structure color provides a powerful strategy to soft robotic areas.

The mechanism of schemochrome changing of CNCs during actuation is shown in Figure 5a,b. When the film is exposed to moisture, the CNCs layer begins to absorb water and swell, which has resulted in the enlargement of the pitch “*p*” of the CNCs cholesteric structure.^[^ ^44^, ^45^ ^]^The film bends towards the side insensitive to water and increases the angle of incidence “*θ*”. In addition, *n*_avg_ decreases because *n* values of CNCs and water are 1.54 and 1.33 respectively, which further induces the differences in reflection for samples. (Figure S12, Supporting Information) Conversely, when the NIR laser light is applied, the CNCs layer starts to dry out, because of the high photothermal conversion efficiency and thermal conductive properties of AgNPs. At the same time, thermal expansion of the PU layer and shrinking of the CNCs layer lead to the diminution of “*θ*”, while the loss of water leads to an increase of *n*_avg_. The change of selectively reflected light of the cholesteric LC conforms to the Bragg's reflection equation of crystal diffraction. As shown in Figure 5c,d, the relationship between the color change and the actuating angle has been studied quantitatively. During the actuating process, the actuator shows a range of angle‐related structural colors including blue (0°), baby blue (5°), green (10°), olive (15°), and brown (>20°).

Inspired by the touch‐sensitive property of mimosas, a various multi stimulus‐responsive biomimetic robot was shaped (Figure 5e,f, Movie S6, Supporting Information). We could prompt the mimosas robot to perform reversible “shy” actions including “roll up” and “stretch out” in a short moment (humidity: 9 s; NIR light: 32 s), which was accompanied by periodic vivid structural color‐changing from brown to green to blue. The comparison of our material and recently reported color‐changing materials confirms that our strategy has great superiority in both the actuating speed and the color‐changing range (Table S1, Supporting Information).^[^ ^20^, ^21^, ^46^, ^47^, ^48^, ^49^, ^50^, ^51^ ^]^

## 3 Conclusion

In summary, inspired by animal skin and butterfly wings, we present a strategy to fabricate multi‐stimuli responsive materials with color‐ and morphing‐change abilities. Benefiting from the CNCs‐based cholesteric LC structure with high water absorption and expansion capacity, extremely low CTE, and linear birefringence, the bilayer actuator exhibits an excellent variable chiral optical property and humidity/NIR light bidirectional reversible actuating performance. In addition, the glucose‐assisted self‐assembly strategy of CNCs endows the actuator with a uniform optical appearance and long‐term stable actuating ability. This work not only offers a feasible approach to realize multiple stimulus responses and simulation of butterflies’ brilliant structural colors at the same time, but also demonstrates the new strategy to realize the visualization of flexible CNCs‐based actuator movement, which could enable further advances in flexible robots, anti‐counterfeiting and information storage fields.

## 4 Experimental Section

### Materials

Cotton cellulose was subjected to cutting and shredding processes and then used without further purification or bleaching. Silver nitrate (AgNO_3_, AR, ≥99.80%), ammonium hydroxide (NH_3_ H_2_O, GR, 25.00–28.00 wt%), glucose anhydrous (AR), analytical grade sulfuric acid (H_2_SO_4_, AR, 95.00–98.00 wt%), and polyvinyl pyrrolidone K30 (AR) were purchased from Chengdu Kelong Chemical Reagent Company (China). Waterborne PU was purchased from Bayer Co., LTD (China). All reagents and solvents were used directly without further purification.

### Preparation of the AgNPs Suspension

AgNPs were prepared by in situ reduction of silver ammonia by glucose. First, 0.20 g of glucose AR, 0.40 g of PVP (2 mol), and 100 mL of deionized water were added into a 250 mL beaker, followed by vigorous stirring for 1 h at 60 °C. Second, 0.14 g of AgNO_3_ was dissolved in 10.00 mL deionized water, then NH_3_ H_2_O was gradually dropped in until the color of the solution changed from transparent to brown and finally back to transparent. Then silver ammonia solution was slowly dropped into the glucose/PVP solution, followed by vigorous stirring for 2 h at 60 °C. Finally, the as‐prepared AgNPs were centrifuged and then resuspended with 20 mL of water, and the concentration of AgNPs suspension was 0.45 wt%.

### Preparation of the PU/AgNPs Layer

First, 5.00 g of PU latex and 5.00 g of AgNPs suspension were added into a 25 mL beaker and mixed well, and bubbles were removed with a vacuum pump. Second, the mixture was added into a Teflon mold and dried at 65 °C for 3 h. Finally, the dried film was taken out, washed with deionized water, and dried at 65 °C for 1 h. The thickness of the elastomer layer maintained a constant value of 0.07±0.01 mm.

### Preparation of CNCs/PU/AgNPs Bilayer Actuator

CNCs were prepared by controlled acid hydrolysis of cotton fibers according to the previous studies. First, the prepared CNCs suspension (1.39 wt%) was concentrated through stirring and water bath heating at 70 °C, then 0.10 g of glucose AR was sufficiently dissolved in 5.00 g of the high concentration CNCs suspension (8.34 wt%). The upper surface of the PU/AgNPs film was coated with a layer of CNCs@glucose mixture. The CNC@glucose suspension with an appropriate viscosity can uniformly distribute across the substrate, and its flow was limited with tape at the boundary. Therefore, the thickness of the CNCs layers can be regulated by changing the suspension mass per unit area of the substrate. To achieve a proper thickness (0.07±0.01 mm), 0.125 grams per square centimeter would be an appropriate amount. Finally, it was dried slowly at room temperature for 48 h. Derived from this as‐prepared bilayer actuation film, several facile bionic 3D actuators were made, such as Rangoon creeper flower, palm, and mimosa, by straightforward cutting and pasting.

### Characterization

SEM (JSM‐5900LV, Japan) samples of the cross‐sections of the CNCs@glucose layer were obtained by directly breaking it into liquid nitrogen with tweezers, then samples were sputter‐coated with gold before imaging. SEM samples of Ag nanoparticles were obtained by drop‐casting 10.00 µL of 20 times diluted AgNPs suspension on to the substrate, then samples were vacuum freeze‐dried before imaging. The infrared thermal imaging device (Fluke Ti70, Everett, WA) was performed to detect the temperature distribution of the samples when NIR laser irradiation was applied and removed. Optical microscopy was performed on a regular digital camera. Laser confocal microscopy Raman spectroscopy (HORIBA, HR Evolution, Japan) with a 785 nm laser line was performed to characterize metal‐ligand interactions. The alterable structure colors of the cholesteric LCs were tested by colorimeter at different view angles. UV‐visible spectrum was performed on a UV‐visible spectrophotometer (MAPADA, P4PC, China).

### Statistical Analysis

The experimental results for SEM images, POM images, Raman microspectroscopy, UV‐visible spectrum, and digital pictures were shown as raw data and no data pre‐processing and statistical analysis have been used. All the dimension data were expressed as mean ± standard error.

## Conflict of Interest

The authors declare no conflict of interest.

## Supporting information

Supporting Information

Supporting Movie 1

Supporting Movie 2

Supporting Movie 3

Supporting Movie 4

Supporting Movie 5

Supporting Movie 6

## Data Availability

Research data are not shared.
