# Supplementary material for: Bioinspired Multi‐Stimuli Responsive Actuators with Synergistic Color‐ and Morphing‐Change Abilities
Source: Adv Sci (Weinh). 2021 Jun 10;8(16):2101295. doi: 10.1002/advs.202101295 (PMC8373155; doi:10.1002/advs.202101295)
Supplement: Supplementary file 1 — Supporting Information [file ADVS-8-2101295-s006.pdf]

WILEY-VCH

© 2021 Wiley-VCH GmbH

ADVANCED SCIENCE
Open Access

Supporting Information

for *Adv. Sci.*, DOI: 10.1002/advs.202101295

Bioinspired Multi-Stimuli Responsive Actuators with Synergistic Color- and Morphing-Change Abilities

Xinkai Li, Jize Liu, Dongdong Li, Shaoquan Huang, Kai Huang and Xinxing Zhang**

Supporting Information

Bioinspired Multi-Stimuli Responsive Actuators with Synergistic Color- and Morphing-Change Abilities

Xinkai Li, Jize Liu, Dongdong Li, Shaoquan Huang, Kai Huang and Xinxing Zhang**

X. Li, J. Liu, Prof. X. Zhang
State Key Laboratory of Polymer Materials Engineering, Polymer Research Institute of Sichuan University, Chengdu 610065, China
E-mail: xxzwwh@scu.edu.cn

S. Huang, Dr. K. Huang
National Engineering Research Center for Non-Food Biorefinery, Guangxi Key Laboratory of Bio-refinery, Guangxi Academy of Sciences, 98 Daling Road, Nanning, 530007, China.
E-mail: hwkai@gxas.cn

D. Li
Guangxi Beitou Environmental Protection&Water Group Co., Ltd. 153 Minzu Avenue, Nanning, 530029, China.

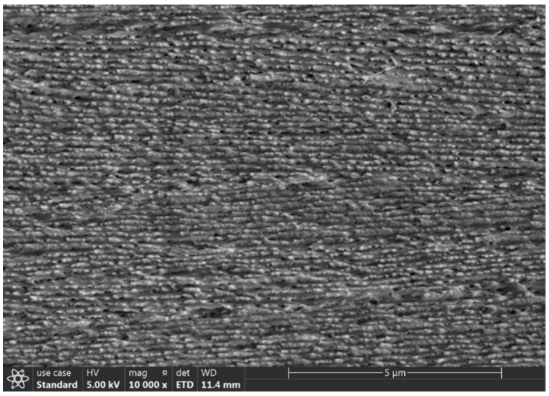

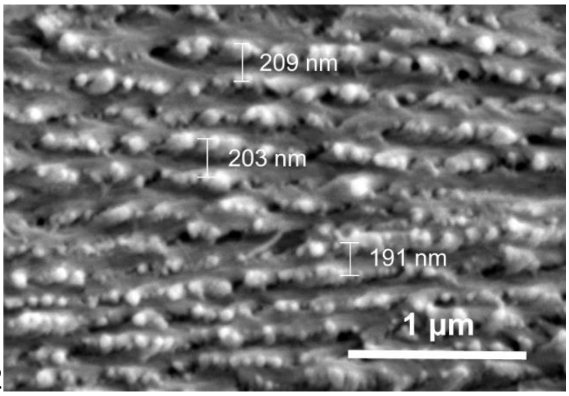

Figure S1. SEM cross-section images of the glucose-assisted assembled CNCs film. The value of p/2 for the CNCs/glucose composite membrane is 201±7 nm.

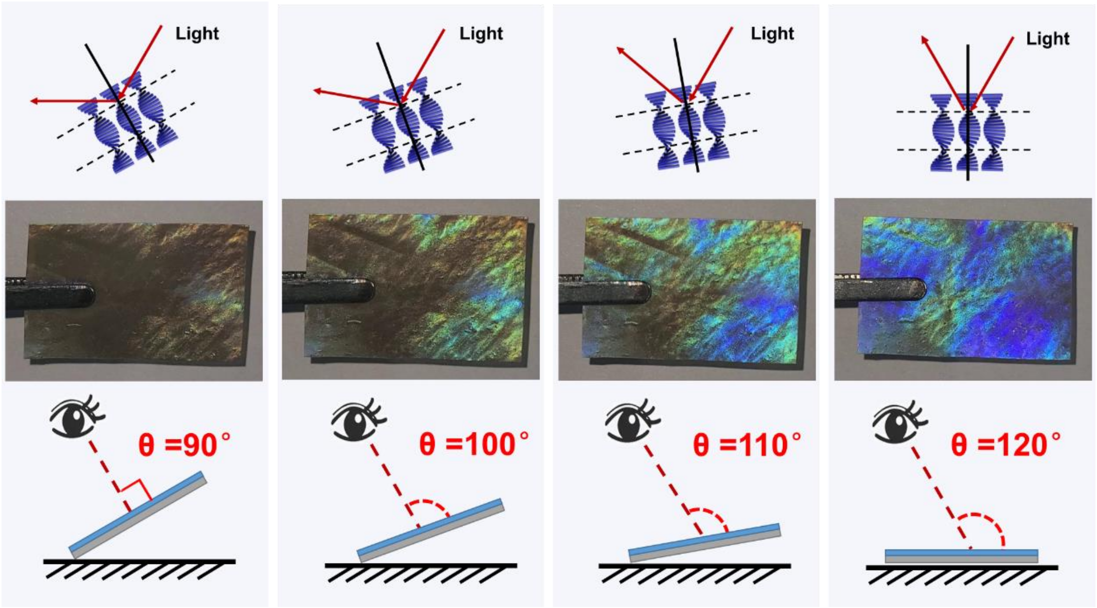

Figure S2. Digital pictures of the cholesteric LC film as the viewing angle increases from 90° to 120°.

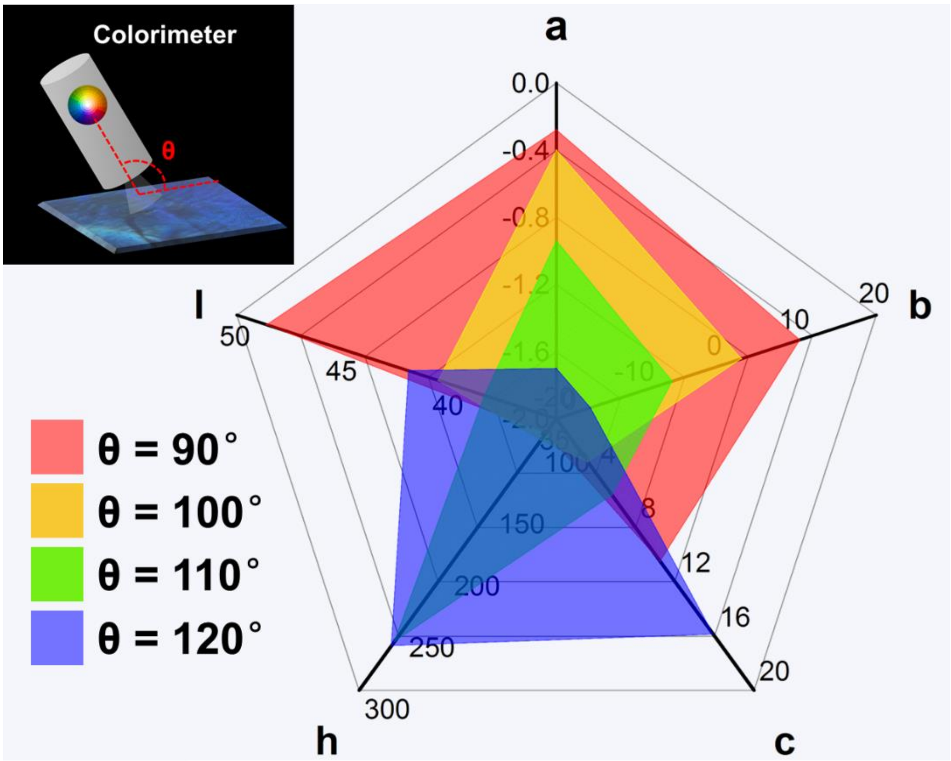

Figure S3. The alterable structure color of the cholesteric LC can be characterized by colorimeter at different view angles (90°, 100°, 110°, 120°).

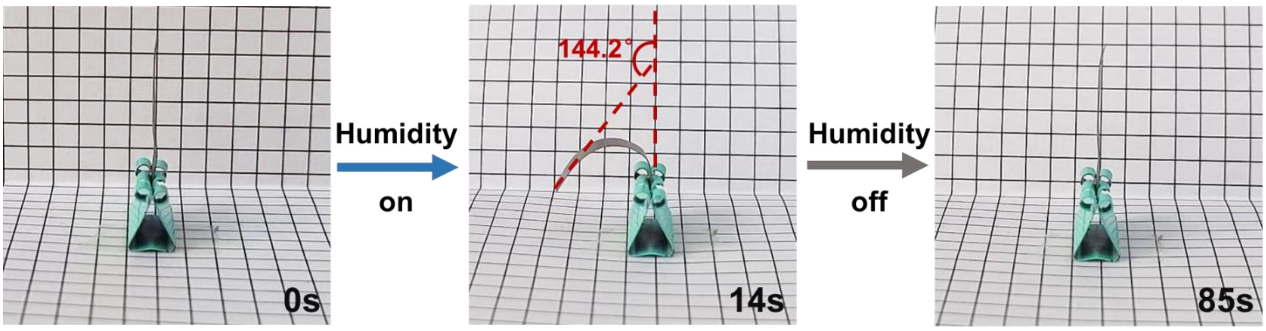

Figure S4. Digital pictures of an actuator reversibly bending in the vertical orientation when humidity is switched on and off. The vertical bending angle of the actuator increases from 0° to 144.2° as the moisture absorption time increases from 0 to 14 s, and then it takes 71 s to revert to its original position without humidifying.

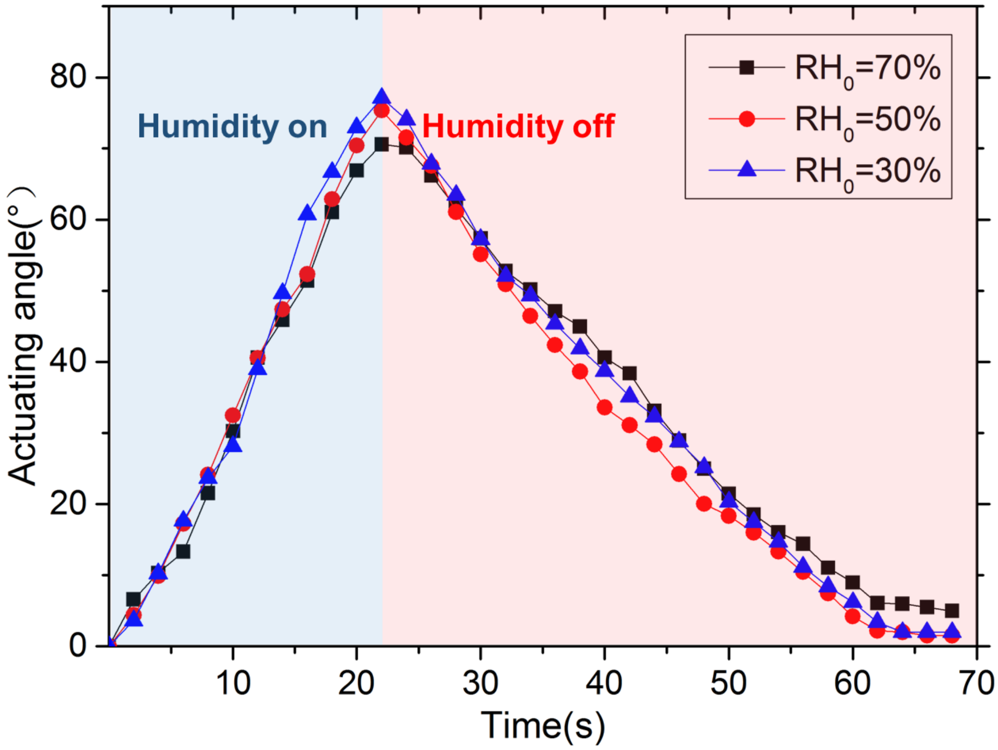

Figure S5. Humidity-actuating performance of the actuator under different original ambient relative humidity (RH) (70%, 50%, 30%). Temperature and the applied additional moisture remain constant.

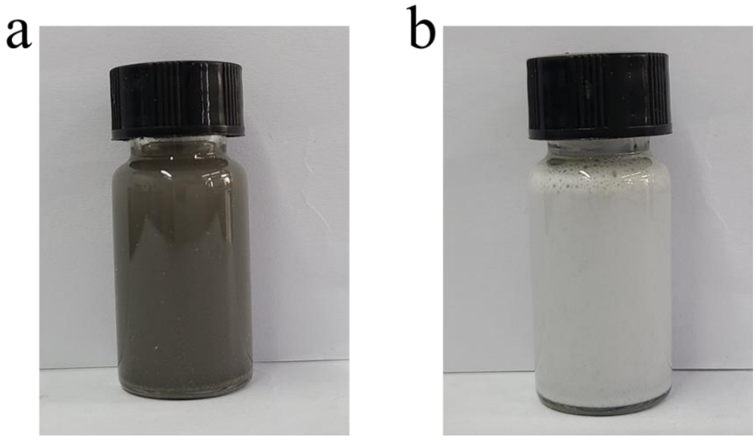

Figure S6. Digital pictures of a) AgNPs aqueous dispersion and b) PU/AgNPs mixture.

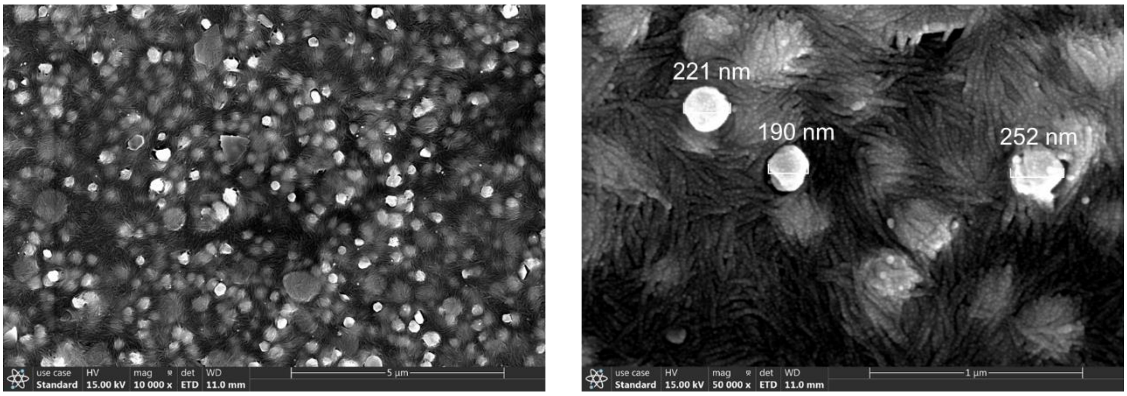

Figure S7. SEM images of the sliver nanoparticles. The value of diameter for the AgNPs is 221±25 nm.

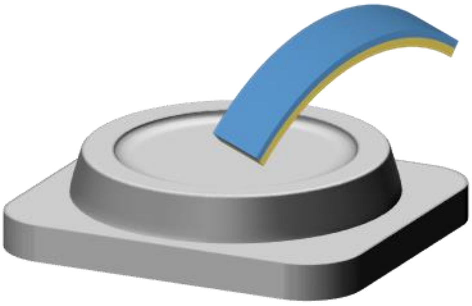

Figure S8. Schematic depiction of the experimental setup used for testing the actuation force of the actuating film.

To confirm photo-induced mechanical stress, we firstly utilize electromagnetic gauge transducer to measure the light-generated force of the actuator films by applying NIR light (808 nm) to a strip-shaped sample ($20 \times 5 \times 0.153$ mm^3) at room temperature. The mechanical force generated by the sample film can be monitored from the following formula: $F = mg$, where m is the real-time mass on indicator of the balance, g is the acceleration of gravity. Then, the mechanical forces are converted into stress followed by the following formula: $P = F/S$, where S is cross sectional area of the strip-shaped film.

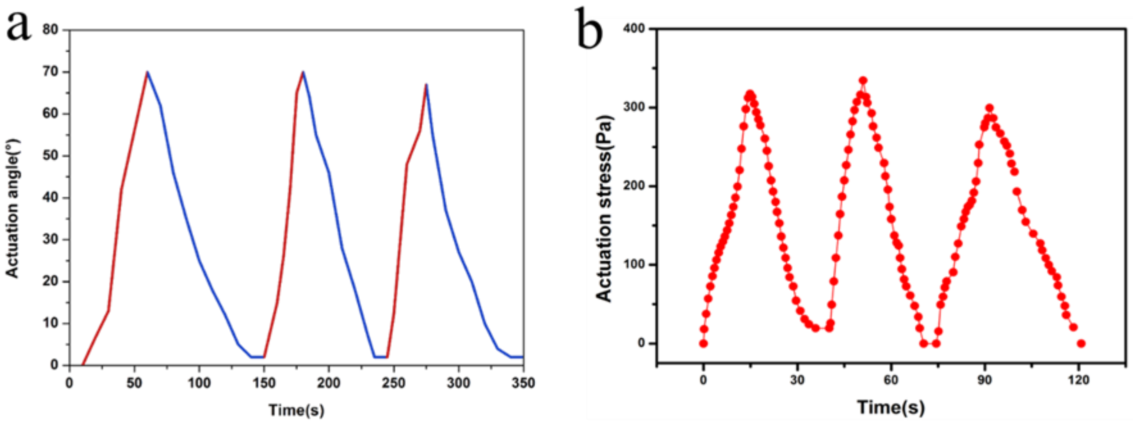

Figure S9. a) Humidity actuation angle and b) actuation stress as a function of time. The cycle of force up and down with humidity on and off, respectively, can be repeated for 3 times without damping.

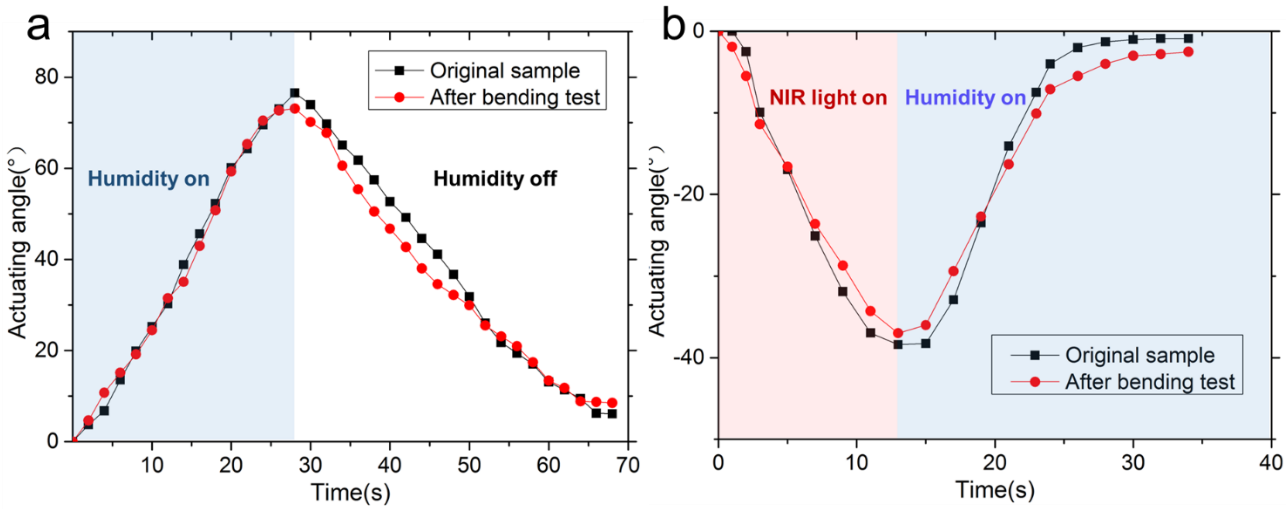

Figure S10. a) Humidity- and b) NIR-actuating performance of the actuator before and after subjected to 5,000 bending cycles.

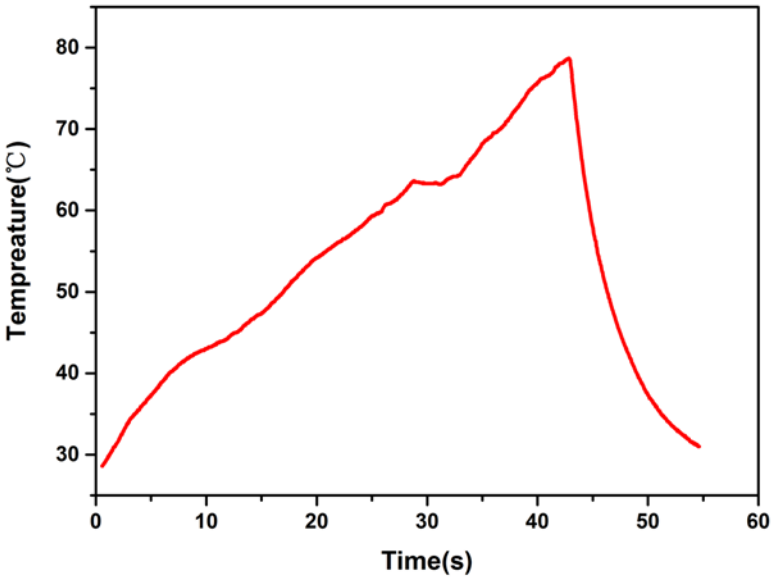

Figure S11. The temperature variation during the NIR-actuation process.

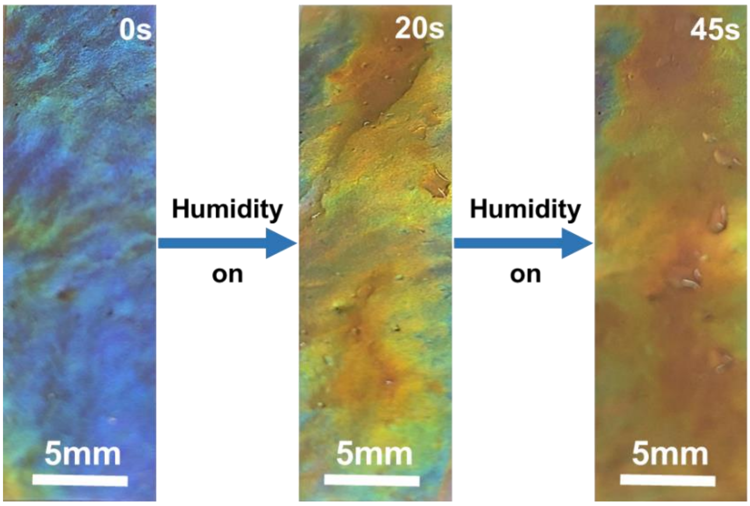

Figure S12. Digital images for structural color change of the CNCs/glucose composite under relative humidity 90%.

Table S1. Comparison of color changing performance of the recently reported materials.

**Ref.**	**Materials**	**Conditions**	**Time**	**Colors**	**Other functions**
[1]	Eu^{3+}-poly(N-isopropylacrylamide) - potassium 6-acrylamidopicolinate	Tb^{3+}	1800s	Red – Green	Actuation
[2]	zinc-ion-intercalated layered polydiacetylene- cellulose paper (T-paper)	40 °C to 140 °C	20 s	Blue – Purple	Actuation
[2]	PI tape-T-paper-MXene/ graphene	40 °C to 140 °C	20 s	Green – Orange	Actuation, Self-sensing
[3]	$Sr_2P_2O_7$:Eu,Y	Strain：20 %-100 %	/	Blue – Purple	/
[4]	Au nanoparticles-poly(N-isopropylacrylamide)	24 °C to 50 °C	/	Red - Grayish violet	/

[5]	single-wall carbon nanotube-line elastomer	NIR irradiation	15 s	Cyan – Blue	Actuation
[6]	liquid crystalline networks-PA	Humidity	6 s	Dark green – Green	Actuation
[7]	P(AAm-co-AAc) - poly(N-isopropylacrylamide)	0 °C to 50 °C	80 s	Green – Red	Actuation
		NIR irradiation	30 - 240 s	Green – Red	Actuation
[8]	Fe_3O_4@PVP-CNCs-poly(N-isopropylacrylamide)	10 °C to 35 °C	/	Green – Yellow – Red	/
This work	CNCs@glucose-PU/AgNPs	Humidity	9 s	Blue – Green – Brown	Actuation
		NIR irradiation	16 s	Brown – Green – Blue	Actuation

Reference

[1] S. Wei, W. Lu, X. Le, C. Ma, H. Lin, B. Wu, J. Zhang, P. Theato, T. Chen, *Angew. Chemie Int. Ed.* **2019**, *58*, 16243.

[2] H. Zhao, R. Hu, P. Li, A. Gao, X. Sun, X. Zhang, X. Qi, Q. Fan, Y. Liu, X. Liu, M. Tian, G. Tao, L. Qu, *Nano Energy* **2020**, *76*, 104926.

[3] B. Tian, Z. Wang, A. T. Smith, Y. Bai, J. Li, N. Zhang, Z. Xue, L. Sun, *Nano Energy* **2021**, *83*, 105860.

[4] A. Choe, J. Yeom, R. Shanker, M. P. Kim, S. Kang, H. Ko, *NPG Asia Mater.* **2018**, *10*, 912.

[5] S. Banisadr, J. Chen, *Sci. Rep.* **2017**, *7*, 17521.

[6] R. Lan, Y. Gao, C. Shen, R. Huang, J. Bao, Z. Zhang, Q. Wang, L. Zhang, H. Yang, *Adv. Funct. Mater.* **2021**, 2010578.

[7] Z. Zhang, Z. Chen, Y. Wang, J. Chi, Y. Wang, Y. Zhao, *Small Methods* **2019**, *3*, 1900519.

[8] H. Ma, M. Zhu, W. Luo, W. Li, K. Fang, F. Mou, J. Guan, *J. Mater. Chem. C* **2015**, *3*, 2848.
